# Concurrent phyllodes tumor, eccrine carcinoma, and multinodular goiter 20 years after radiotherapy for Hodgkin lymphoma

**DOI:** 10.1002/ccr3.1854

**Published:** 2018-11-06

**Authors:** Kristen M. Quinn, Gary Tozbikian, William E. Carson

**Affiliations:** ^1^ Division of Surgical Oncology The James Cancer Hospital at The Ohio State University Columbus Ohio

**Keywords:** breast cancer, eccrine adenocarcinoma, phyllodes tumor, radiotherapy

## Abstract

This unusual case of concurrent eccrine adenocarcinoma, phyllodes tumor, and multinodular goiter serves to alert the oncologic community to the high prevalence of second cancers after childhood radiotherapy. Increased surveillance and index of suspicion are recommended to successfully diagnose and treat second primary cancers in this vulnerable population.

## 1 INTRODUCTION

We present a patient with a history of Hodgkin lymphoma as a teenager, who later developed phyllodes tumor, cutaneous adnexal carcinoma with eccrine differentiation, and multiple thyroid nodules. This case details the presentation, diagnosis, and treatment of these rare tumors in a childhood cancer survivor.

Due to modern therapeutic advances, the majority of patients with childhood Hodgkin lymphoma (HL) are able to achieve long‐term survival; however, late complications (namely cardiovascular or pulmonary) and second cancers have emerged as culprits of early morbidity and mortality in these patients. Survivors of HL have a solid tumor cancer risk over twofold the general population. Large combined analyses show that after 10 years of first treatment, lung cancer accounts for the largest absolute excess cancer risk (34/10 000 HL patients per year), however in women, it is breast cancer (40/10 000 HL patients per year).^1^, ^2^, ^3^ Prognosis for patients after the diagnosis of a second cancer is poor; Ng et  al^4^ report a 38.1% 5‐year survival. Efforts to minimize carcinogenic chemotherapy regimens and radiation fields have seen some success, but additional research is needed to appropriately screen and diagnose second cancers in the population currently decades post‐therapy. Some studies have examined the median time to onset of solid cancer tumors, finding an average time of onset of 12.2 years.^5^ The patient presented here developed a second and third cancer 20 years after treatment for childhood HL. This case report serves to detail the diagnosis and treatment of her unusual malignancies, as well as support the growing body of evidence documenting the additional risks and recommended surveillance for survivors of childhood cancer.

## 2 CASE PRESENTATION

A 35‐year‐old Caucasian female patient presented to her local emergency department in November 2016 with a chief complaint of neck pain. The patient had a past medical history significant for Hodgkin lymphoma diagnosed in 1998 following excision of a neck mass at age 16. She underwent chemotherapy and mantle field radiation in 1998. The radiation targeted lymph nodes in the neck, axilla, and behind the sternum in order to encompass the nodal basin of her cancer and the common lymph node drainage areas. The patient denied any history of radiation to her face. She reported remission at the time of presentation for this complaint of neck pain and was not following with anyone for her history of HL. She had no notable past surgical history. Menarche was at age 13 and she gave birth to one child at age 18. The patient’s family history was unremarkable with the exception of ovarian cancer in her maternal great aunt. There was no family history of breast or thyroid cancer. The patient was a previous smoker, quitting after about 10 years of use. No drug or alcohol use was recorded.

In the emergency department, a neck CT revealed a subcutaneous mass over the mid‐clavicle, a breast mass, and multiple nodules in the thyroid gland with the largest nodule measuring 1.5 × 1.6 × 2.0 cm. The breast mass had dimensions of 2.6 × 4.0 × 4.9 cm by ultrasound. The patient was instructed to follow‐up in breast and thyroid clinics for these findings.

The patient followed the emergency department’s recommendations and was examined by a surgical oncologist. In the breast clinic, she stated that the large right upper‐outer quadrant breast mass had been present for 1 year. She was unsure how long the mass overlying the clavicle had been present, as it had been asymptomatic. On physical examination, the patient appeared well developed and well nourished. Respiratory, abdominal, musculoskeletal, and cardiovascular systems were normal. An 8.0 cm mass was located in the upper‐outer quadrant of the right breast centered at the 10:00 axis about two fingerbreadths on the nipple border. Nipples were normal bilaterally. There was no cervical, supraclavicular, or axillary lymphadenopathy. Directly overlying the clavicle about two fingerbreadths medial to the mid‐clavicular line was a 0.6 cm mobile mass within the skin. It was not associated with any regional lymphadenopathy. Laboratory workup was negative and unremarkable.

**Figure 1:**
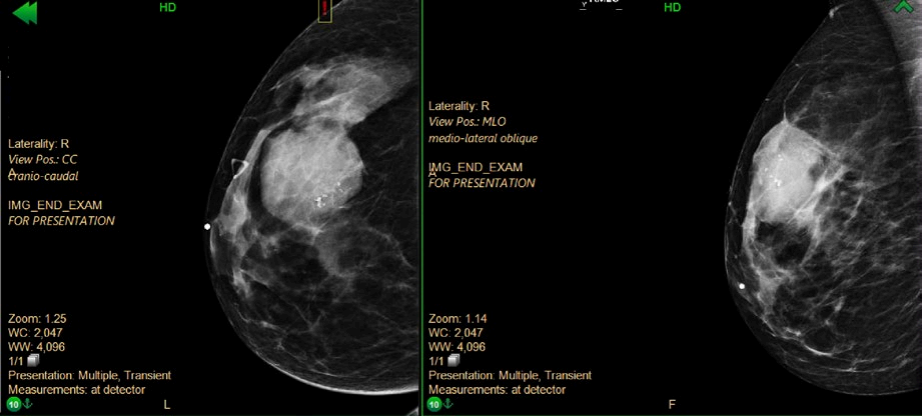
Mammogram of right breast, cranio‐caudal and medio‐lateral oblique views, respectively

**Figure 2:**
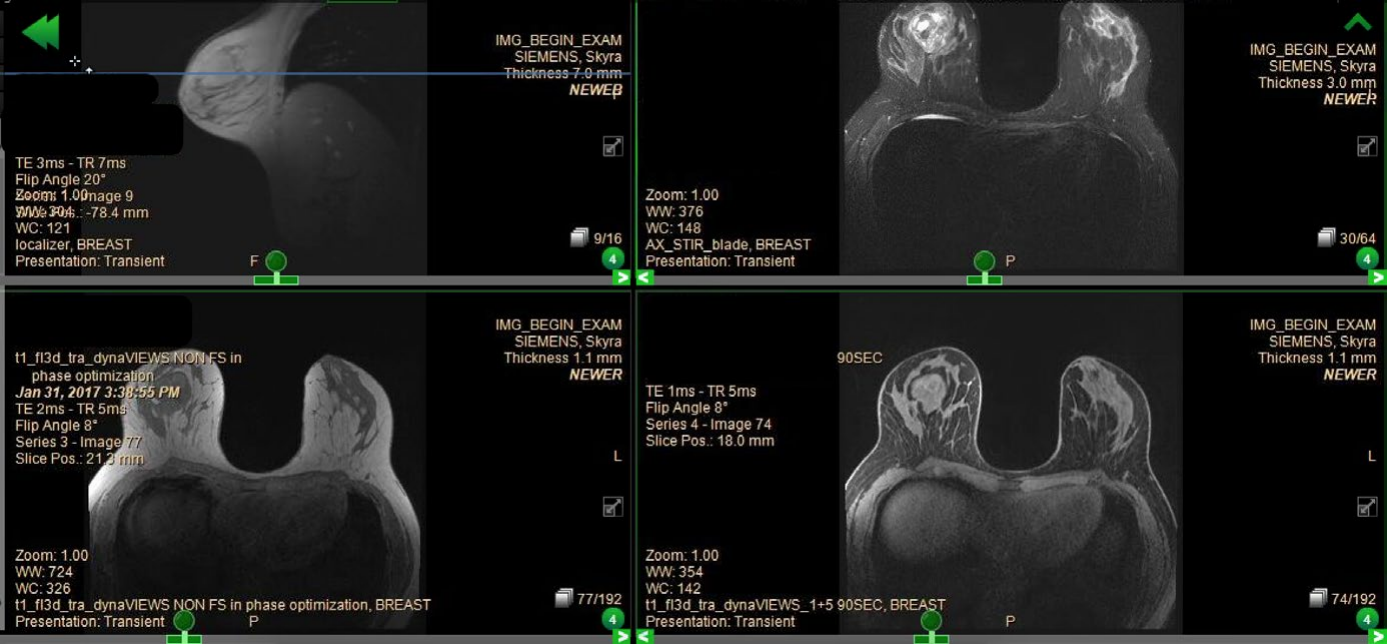
MRI of breast showing a large right breast mass

The patient underwent a bilateral mammogram in December 2016, followed by ultrasound‐guided core biopsy of the breast mass. Initial core biopsy performed at an outside institution of the right breast mass came back as fibrocystic change. The outside biopsy was not reviewed at our institution. Based on a high level of clinical suspicion, additional imaging and a repeat biopsy were performed at our institution in January 2017. The repeat biopsy of the right upper‐outer quadrant breast mass showed a phyllodes tumor. Pathology results described a fibroepithelial lesion with hypercellular stroma, mild‐moderate stromal cytologic atypia, increased stromal mitotic activity (4‐5/10 HPF), and focal areas suggestive of phyllodes architecture. The nature of the margins (pushing or infiltrative) could not be determined from the biopsy material. MRI showed the phyllodes tumor in the right breast measuring 4.7 cm. An excisional biopsy of the clavicular mass was done in a separate operation. The biopsy result was a cutaneous adnexal adenocarcinoma with eccrine differentiation. Surgical excision was recommended for both the breast and clavicular masses (Figures 1 and 2).

The patient also followed up in thyroid clinic for the multinodular goiter seen on her CT scan of the neck in the emergency department. Review of systems in the thyroid clinic was negative for change in voice or positional dyspnea but was significant for difficulty swallowing that started roughly 3 months prior. The patient also had pain in the right lower neck. She described the pain as constant, with an intensity of 5/10, and alleviated by acetaminophen. Ultrasound revealed three complex nodules with the largest in the left lobe measuring 1.3 × 1.8 × 2.5 cm, and other smaller nodules. The patient was diagnosed with multinodular goiter at this time. Two nodules met criteria for FNA. Cytology for both nodules was benign. The patient elected to defer any intervention and did not continue to follow‐up.

In early March 2017, the patient underwent wide local excision of the phyllodes tumor, wide local excision of the cutaneous adnexal adenocarcinoma and right axillary sentinel lymph node biopsy, and concurrent post‐reduction bilateral oncoplastic reconstruction. Surgery entailed intra‐dermal injections of Tc99m‐filtered sulfur colloid 1‐2 cm from the margins of the lesion located over the right clavicle. Lymphoscintigraphy revealed uptake in two right axillary nodes. Once in the operating room, a standard axillary incision was made and 2 “hot” and blue lymph nodes were identified and removed. The cutaneous adnexal adenocarcinoma was then resected with a 1.5 cm margin which created a 4 × 10 cm ellipse. A lumpectomy was performed through predesigned incisions to ensure a cosmetically favorable closure for the phyllodes tumor in the right upper‐outer breast. After removal, the plastic surgery team completed a bilateral breast tissue rearrangement and left breast reduction for symmetry. All aspects of the operation went smoothly, and the patient recovered uneventfully.

**Figure 3:**
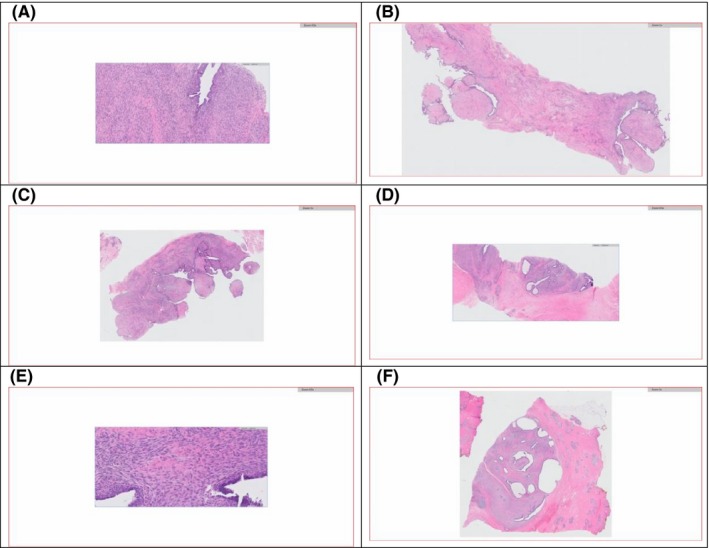
H&E‐stained biopsy results. A, Core needle biopsy, 10×, increased stromal cellularity. B, Core needle biopsy, 1×, epithelial‐lined stromal projections protrude into cystic spaces. C, Core needle biopsy, 1×, increased stromal cellularity, stromal cuffing, epithelial‐lined stromal projections protrude into cystic spaces. D, Core needle biopsy, 2×, heterogeneity in stromal cellularity. E, Core needle biopsy, 20×, increased stromal cellularity, 4‐5 mitoses per 10 high powered fields. F, Excision, 1×, circumscribed borders, increased stromal cellularity, epithelial‐lined stromal projections protrude into cystic spaces

Pathology confirmed a phyllodes tumor measuring 4.1 cm in greatest diameter and clear margins. The phyllodes tumor pathology showed a circumscribed border, mild to moderate stromal cellularity, mild stromal cytologic atypia, and a mitotic rate of 4‐5/10 HPF. Necrosis and malignant heterologous elements or stromal overgrowth were not identified. Overall, features were consistent with a benign phyllodes tumor (Figure 3).

The adnexal neoplasm in the right chest was resected with negative surgical margins, and 0 of 2 nodes were positive for metastatic disease. The pathology report noted the presence of mitotic figures and rare atypical mitotic figures, favoring the diagnosis of a malignant adnexal neoplasm. The report adds that since the breast is a modified sweat gland, it is impossible to distinguish a primary cutaneous adnexal neoplasm from a primary breast neoplasm based on histologic features and that no immunoperoxidase stains can distinguish these two entities.

On her first postoperative clinic visit, the patient was recovering well. Her incisions were clean, dry and intact without erythema, drainage, hematoma or seroma. The patient has since continued to follow‐up and has not experienced any complication or recurrence. She is recommended to follow‐up annually.

## 3 DISCUSSION

Hodgkin lymphoma (HL) accounts for approximately 7% of childhood cancers and is the most common childhood cancer in the 15‐19‐year‐old age‐group.^6^ Most children and adolescents with HL have an excellent prognosis, with high‐risk disease patients reaching cure rates above 84% with modern therapy.^7^ However, the standard treatment for HL which includes chemotherapy and radiotherapy carries significant risk. These patients continue to receive additional imaging and radiation exposure in order to monitor the clinical response to treatment and survivorship surveillance. Acute radiation effects such as erythema, hyperpigmentation, dry mouth, granulocytopenia, or thrombocytopenia are well documented. These effects are typically self‐limited and reversible; however, the long‐term effects, especially in adolescent patients, are less clear.

The Childhood Cancer Survivor Study funded by the National Cancer Institute identified a number of potential late effects from radiation, including premature menopause, stroke, and second cancers.^8^ Here, we present a case of multiple concurrent second cancers that developed 20 years after a patient was treated for Hodgkin lymphoma at age 16. While secondary neoplasms in survivors of childhood HL are not common, they are also not rare. Large cohort and population‐based studies have shown that survivors of childhood HL have the highest cumulative incidence of secondary neoplasms (nearly 30% at 25 years after) among patients with childhood diagnosed primary malignancies.^9^, ^10^, ^11^ Some studies report as high as an 18.5‐fold increased risk of developing a second cancer in HL patients compared to the general population.^12^ In this series, the most common secondary malignancies included breast cancer, thyroid cancer, acute myeloid leukemia, and soft tissue sarcomas, with breast cancer incidence directly relating to the dose of radiation delivered to the chest.^12^ Due to the significant risk of breast cancer, current guidelines recommend that survivors of childhood cancer treated with chest radiation undergo annual screening mammography starting at age 25.^13^ Despite these recommendations, Oeffinger et  al^14^ studied a cohort of 1976 women who had childhood cancer treated with chest radiation and found that 63.5% of women aged 25‐39 had not received a screening mammogram within the past 2 years.

Many survivors of childhood cancer (~5%) may develop three or more secondary malignancies. The patient reported here presented 19 years after treatment for her HL with multiple second cancers, one of which was a phyllodes tumor (PT) of the breast. PTs are rare fibroepithelial tumors of the breast, accounting for 0.3%‐1% of all breast tumors and are even less common in persons younger than 40.^15^ PTs are classified into benign, borderline, and malignant. Benign PT has a similar appearance on cytology to the common fibroadenoma. However, histologic features, particularly the presence of mitoses and increased stromal cellularity, can help distinguish a benign PT from fibroadenoma on core needle biopsy specimens.^16^ Treatment is surgical resection with 1 cm margins.^17^ Thorough literature searches found the combination of this rare tumor after successful treatment for HL reported in only one case in Morocco.^18^ A concurrent phyllodes tumor was detected in an adolescent as part of her 18 F‐FDG PET/CT imaging during therapy for Hodgkin lymphoma, perhaps suggesting there could be a role for 18F‐FDG PET/CT in the prediction of local recurrence or distal metastases of malignant phyllodes lesions.^19^ Widening the literature search to include phyllodes tumor after radiation for any kind of malignancy yielded two more cases.^20^, ^21^ These studies in combination suggest that the malignant transformation of a fibroadenoma or the development of a PT as a second cancer must be considered in all patients who have received chest radiation for any cause.

Few cases have described phyllodes tumors associated with other tumors. Of those reported, invasive ductal carcinoma is most often found in the same or other breast.^22^, ^23^ Some studies have grouped these cases and determined the presence of a ductal carcinoma was not indicative of a worse prognosis, but rather an incidental finding.^24^ Postoperative pathology after mastectomy has even shown foci of invasive carcinoma arising within the phyllodes tumor, and, in one study, multiple adjacent fibroadenomas.^25^ Overall, the relationship between PTs and other independent concurrent breast lesions remains unclear. Thus, we felt this case presented an opportunity to alert physicians to the high incidence of second cancers seen in adulthood after treatment for childhood cancer, document the unusual coexistence of two concurrent primary cancers in an irradiated field, and reinforce the high index of suspicion needed to diagnose PTs early.

The patient also had a clavicular mass which proved to be an adnexal cutaneous tumor. Adnexal carcinomas are rare among cutaneous tumors, making prognostic factors difficult to elucidate.^26^ They are classified according to their differentiation toward one of the appendageal skin structures (eccrine, apocrine, follicular, or sebaceous). Carcinomas of eccrine derivation are commonly called sweat gland tumors and can be benign or malignant. Treatment is surgical excision, though some patients may receive surgery plus radiation.^26^

These two rare tumors were found simultaneously in a previously radiated field. The incidence of radiotherapy‐induced malignancies has been increasing, especially in survivors of pediatric cancers as a result of therapeutic improvements. However, the pathophysiology of this trend has not been clearly elucidated. Intuitively, the tumorigenic effects of ionizing radiation are increased with younger age of exposure, as this allows more time for mutation and malignancy to develop. The genetic background of the patient in this report could have potentially harbored undetermined germline mutations in tumor suppressor genes, putting her at risk of cancer as a pediatric patient and second cancers. Survivors of Hodgkin lymphoma are at greatest risk of developing breast cancers after chest radiotherapy.^27^ In fact, breast cancer risk was increased in all survivors of childhood cancer who were treated with chest radiation therapy, with a standardized incidence ratio of 24.7 according to Kenney et  al.^28^ Additional significant independent risk factors for developing a second cancer include family cancer history and a history of thyroid disease. Though asymptomatic, it is interesting that this patient was found to have multinodular goiter. However, up to 5% of patients will have a negative family history, no radiation, and no chemotherapy before developing a second primary malignancy. Therefore, although most cases can be attributed to radiation, genetic disease, chemotherapy, or a combination of these, unrecognized predisposition or chance must also play a role.

Kadan‐Lottick et  al explored not only the future risk of pediatric cancer survivors, but also evaluated these patients’ knowledge of their past diagnosis, treatment, and thus their motivation to seek medical attention and follow‐up. Examining over 600 survivors, this group found that only 72% of patients accurately reported their diagnosis with precision.^29^ These findings present an important opportunity for intervention. The better childhood cancer survivors understand their previous diagnosis and therapies, the better they can articulate their future risks to other healthcare professionals, and the more likely they will be to receive appropriate long‐term follow‐up care.

In summary, the study of secondary malignancy after mantle radiation for HL has wide‐ranging implications for both treatment, screening, and early intervention pertaining to second cancers. Obstacles to studying these phenomena include the wide genetic diversity of cancer survivors, differences in tumor histology, and evolving treatment regimens. Additionally, individual differences in access to healthcare and health literacy limit patients’ ability to be their own advocate. A comprehensive understanding of the pathway of radiation‐induced breast or adenocarcinomas can guide changes in the microenvironments that foster the growth of these second cancers. While developing medications to target the pathophysiology of cancer post‐radiation exposure, we can concurrently work to modify and optimize radiotherapy techniques.

## 4 CONCLUSION

This unusual case of concurrent eccrine adenocarcinoma, phyllodes tumor, and multinodular goiter serves to alert the oncologic community to the high prevalence of second cancers after childhood radiotherapy and detail their successful identification and treatment. This report adds to the growing number of documented second cancers in this population and supports early screening and close adherence to recommendations. Monthly breast self‐examination, annual breast examinations by healthcare providers until age 25, and every 6 months thereafter are crucial to early detection. It is the authors’ practice that patients who were treated with radiation therapy before age 30 should be considered for yearly breast MRIs in addition to mammograms beginning at age 25 or 8‐10 years after radiation therapy in accordance with NCCN guidelines.^30^ Further, this report illustrates how asymptomatic phyllodes and eccrine adenocarcinoma present, and the high index of suspicion required to accurately diagnose and treat them. We support greater research to explicate the pathophysiology of radiation‐induced malignancies, so that future surveillance, medical therapies, and safer radiotherapies can be developed and employed in this vulnerable population.

## CONFLICT OF INTEREST

None declared.

## AUTHOR CONTRIBUTION

KMQ: is the main author of the manuscript and primarily conducted the research and writing of this clinical case. GT: contributed his expertise in pathology to this clinical case; provided pathologic imaging and worked to review and edit the manuscript. WEC: contributed to this case as the surgeon and clinician of this patient; provided his surgical oncology expertise to edit and review this manuscript.
